# Predictive potential of optical coherence tomography parameters for the prognosis of decreased visual acuity after trabeculectomy in open-angle glaucoma patients with good vision

**DOI:** 10.1186/s12886-023-03145-3

**Published:** 2023-10-04

**Authors:** Yoko Takeda, Naoki Takahashi, Naoki Kiyota, Taiki Kokubun, Satoru Tsuda, Kazuko Omodaka, Yu Yokoyama, Toru Nakazawa

**Affiliations:** 1 Department of Ophthalmology, Tohoku University Graduate School of Medicine, 1-1 Seiryo-machi, Aoba-ku, 980-8574 Sendai, Miyagi Japan; 2 Department of Retinal Disease Control, Ophthalmology, Tohoku University Graduate School of Medicine, Sendai, Japan; 3 Department of Advanced Ophthalmic Medicine, Tohoku University Graduate School of Medicine, Sendai, Japan

**Keywords:** Risk factors, Optical coherence tomography, Surgical treatment, Visual acuity, Trabeculectomy

## Abstract

**Background:**

Trabeculectomy (trab) is the most effective surgical procedure for lowering IOP and preventing glaucoma progression. However, decline in best-corrected visual acuity (BCVA) is one of the most serious postoperative complications of trab. Here, we investigated methods to predict decreased BCVA after trab in glaucoma patients with good preoperative BCVA.

**Methods:**

This study included 35 eyes of 35 open-angle glaucoma (OAG) patients (male / female: 21 / 14, age: 64.0 ± 9.7 years old, preoperative intraocular pressure: 15.9 ± 5.4 mmHg, mean deviation: -18.1 ± 5.6 dB) with preoperative BCVA of 0.7 or better who underwent trab and were observed for more than 12 months. As a preoperative analysis, we measured temporal quadrant circumpapillary retinal nerve fiber layer thickness (cpRNFLT) and ganglion cell complex thickness in a central strip between the disc and fovea (csGCCT), an area that corresponds to the location of the papillomacular bundle (PMB) in swept-source optical coherence tomography (OCT). We defined BCVA decline as a loss of more than 3 lines of BCVA after 12 months. Measurement parameters were compared between the BCVA-decline group and the non-BCVA-decline group.

**Results:**

BCVA decline was detected in 11 cases (31.4%) 12 months after trab. There was a statistically significant difference in axial length (P = 0.049). A single logistic analysis showed that the BCVA-decline group had significantly lower cpRNFLT than the non-BCVA-decline group (27.7 ± 8.0 μm vs. 45.1 ± 5.3 μm, P < 0.001, cut-off value: 33.4 μm), as well as lower csGCCT (72.4 ± 7.7 μm vs. 87.5 ± 5.1 μm, P = 0.002, cut-off value: 82.3 μm). Multivariable logistic analysis showed that the BCVA-decline group had significantly lower temporal quadrant cpRNFLT (P < 0.001) and lower middle csGCCT (P < 0.001) compared to the non-BCVA-decline group.

**Conclusions:**

Lower temporal quadrant cpRNFLT and middle csGCCT, OCT scan areas that correspond to the location of the PMB, might be biomarkers that predict BCVA decline after trab in OAG patients with good vision.

**Supplementary Information:**

The online version contains supplementary material available at 10.1186/s12886-023-03145-3.

## Background

Open angle glaucoma (OAG) is an important, vision-threatening disease. It is multifactorial [1], with risk factors that have been reported to include high intraocular pressure (IOP), aging, myopia [2], family history [3], reduced central corneal thickness (CCT), corneal hysteresis, low ocular blood flow (OBF), lamina cribrosa abnormalities, oxidative stress, and neuroinflammation, in addition to lifestyle factors, such as diabetes, sleep apnea, diet, and smoking [1, 4–9]. In Asia, normal-tension glaucoma (NTG) is the most common type of open-angle glaucoma [2]. However, even in NTG, lowering IOP is the only evidence-based treatment for preventing glaucoma progression [10]. Clinically, the main treatment method is to use various kinds of eye drops, which have various mechanisms for lowering IOP. When these treatments are not effective for lowering IOP, further treatments, including laser irradiation and surgery, are performed. Ultimately, the purpose of all these treatments is to maintain quality of life (QOL) for patients over their entire lifespan.

Trabeculectomy (trab), first reported by Cairns in 1972, allows about 70% of patients to maintain good control of IOP [11]. It is the most effective surgical procedure for lowering IOP and preventing glaucoma progression. The success of trab depends on the postoperative wound healing process. During healing, the excessive proliferation of fibroblasts in subconjunctival tissues, such as Tenon’s capsule, causes bleb failure [12, 13]. However, the most serious postoperative complication of trab is a decline in best-corrected visual acuity (BCVA). BCVA decline after trab is referred to as the “wipe-out” phenomenon; studies on wipe-out have reported incidences that ranged from 0 to 15% [14–21]. These studies have reported risk factors that included a narrow visual field (confined to a central 10-degree island) [14], older age [16], high mean postoperative IOP, and hypotony maculopathy [19]. BCVA is important for maintaining the QOL of glaucoma patients and, in the clinic, physicians may sometimes hesitate to perform trab because of uncertainty about the risk of serious VA decline, even in cases of advanced glaucoma. Furthermore, BCVA decline after trab also occurs in the mild and middle stages of glaucoma and in patients with good preoperative BCVA; the precise cause is still unknown. This is especially a concern in Asia, because patients with NTG are more susceptible to changes in central vision [22] and are at risk for trab-associated BCVA decline.

Papillomacular nerve fiber bundle (PMB) thickness is closely correlated with BCVA in glaucoma patients. Previously, we developed an original scan pattern and showed its usefulness for the evaluation of visual function in glaucoma patients [23, 24]. Recently, we also confirmed the specific area of the PMB that is related to BCVA changes in the advanced stage of glaucoma [25, 26]. We sometimes observe a discrepancy between structure and function in glaucoma patients when they are young. These patients have a good central visual field and good BCVA, but a thin PMB. However, there might be significant concerns about whether they will be able to maintain their visual function as they age. We consider that treatment strategies for cases where the PMB is thin, yet BCVA is maintained, can be challenging.

We previously reported that low RNFLT in the PMB area was a risk factor for BCVA decline after trab in a retrospective study based on spectral-domain OCT findings [27]. There are potential benefits to knowing that PMB thickness can predict postoperative BCVA decline. Thus, we launched the current study, which was designed as a 12-month retrospective case-control study of OAG patients with relatively good preoperative BCVA. We obtained preoperative SS-OCT measurements of the PMB and determined whether they could be used to predict BCVA decline (defined as a ≥ 3-line change in the logarithm of the minimum angle of resolution (logMAR)). The findings of this study may help to improve decision-making for surgical interventions and minimize the risk of serious BCVA decline after trab.

## Materials and methods

### Subjects

This study enrolled 35 eyes of 35 patients with OAG (male/female: 21/14, age: 64.0 ± 9.7 years, preoperative IOP: 15.9 ± 5.4 mmHg [under anti-glaucoma eye drops and/or oral medication], mean deviation (MD): -18.1 ± 5.6 dB, cpRNFLT: 53.8 ± 14.6 μm) who underwent trab at Tohoku University Hospital between August 2018 and July 2020. The decision to perform trab was made by a glaucoma specialist (T.N.) in reference to the Japan Glaucoma Society Guidelines for Glaucoma, 3rd edition [28]. The inclusion criteria were (1) a diagnosis of OAG, including either primary open-angle glaucoma (POAG), NTG, or pseudo-exfoliation (PE) glaucoma, and (2) a glaucomatous visual field defect, meeting the Anderson-Patella classification, i.e., with one or more of the following: (a) a cluster of three points with a probability of < 5% on the pattern deviation map in at least one hemifield (including ≥ 1 point with probability of < 1% or a cluster of two points with a probability of < 1%, (b) glaucomatous hemifield test results outside the normal limits, and (c) a pattern standard deviation beyond 95% of normal limits, as confirmed in at least two reliable examinations. The exclusion criteria were (1) age younger than 20 years, (2) preoperative decimal BCVA less than 0.7, (3) axial length ≥ 28.0 mm [25, 29, 30], and (4) the presence of other ocular diseases or systemic diseases affecting the visual field. Previous studies have used two lines of loss, or an equivalent change in logMAR, in advanced glaucoma patients who underwent trab [15, 17, 31]. We examined risk factors that predicted BCVA decline after trab, using a strict definition of BCVA decline: three logMAR lines in patients with a decimal BCVA of 0.7 or better, which is the borderline for obtaining a driver’s license in Japan. This study adhered to the tenets of the Declaration of Helsinki, and the protocols were approved by the Clinical Research Ethics Committee of the Tohoku University Graduate School of Medicine (study 2021-1-265).

### The measurements of clinical parameters

The preoperative clinical parameters were age, gender, IOP, CCT, axial length, BCVA, and visual field findings. IOP was measured with Goldman applanation tonometry before trab and 1, 3, 6, and 12 months after trab. To exclude possible bias arising from visual acuity improvement caused by concomitant cataract surgery, patients whose BCVA improved after the surgery were excluded. CCT was measured with anterior-segment OCT (Casia, Tomey Corporation, Nagoya, Japan). Axial length was measured with ocular biometry (OA-2000, Tomey Corporation, Nagoya, Japan). BCVA was measured with a standard Japanese decimal visual acuity chart and converted to logMAR. All the preoperative examination data were obtained within a 2-month period. We examined BCVA and IOP and performed an OCT evaluation. MD and the visual field index (VFI) were measured with the Humphrey visual field analyzer (HFA) using the Swedish interactive threshold algorithm (SITA)-standard strategy of the 24 − 2 program. As in our previous work, we analyzed average total deviation (TD) in the 4 paracentral points, termed TD-central [32]. Only reliably measured data were used (i.e., fixation loss < 20%, false-positive errors < 15%, and false-negative errors < 33%). The LSFG-NAVI device (Softcare Co., Ltd., Fukutsu, Japan) was used to assess optic nerve head-tissue blood flow (ONH-tissue BF) by measuring tissue-area mean blur rate (MT). LSFG images were captured after the subjects rested for 15 min in a dark room. For comparisons of preoperative and postoperative parameters, we collected temporal cpRNFLT and TD-central data from one month to twelve months postoperatively if available. If data from multiple measurements during the postoperative period were available, we selected the data closest to the one-month time point.

### OCT examination

Swept-source OCT (DRI OCT Triton, Topcon Corp., Tokyo, Japan) was used for the assessment of structure. Macular maps (6 × 6 mm, 512 A scans × 256 frames) were obtained and software (FastMap version 10.16, Topcon) was used to determine the thickness of the ganglion cell complex (GCC) layer. Volume scans of the optic disc were also obtained. Total circumpapillary retinal nerve fiber layer thickness (cpRNFLT) and cpRNFLT in the quadrants (superior, nasal, inferior, and temporal) were used for the analysis. The evaluation of the PMB was performed as previously described [25]. Briefly, we used wide scans (12 × 9 mm) and selected a central vertical B-scan (1.5 × 9 mm) that ran through the midpoint of the disc-fovea line, which was automatically detected by the program included in the accompanying software. From this B-scan, we extracted a 1.5 mm × 6.6 mm central strip and divided it lengthwise into three 1.5 mm × 2.2 mm sections. These three sections comprised superior, middle, and inferior central-strip GCCT (csGCCT). This central strip can be considered less susceptible to the effects of the peripapillary chorioretinal atrophy zone than the ONH. All OCT scans with image quality less than 40 were excluded [25].

We included cases that underwent trab with a standard technique by one of three glaucoma specialists (T.N., Y.Y., and S.T.) between August 2018 and July 2020. The surgery was performed in either the superionasal or superiotemporal areas under retrobulbar anesthesia. After formation of a fornix-based conjunctival flap, a scleral flap (4.0 × 3.0 mm) with approximately half the thickness of the sclera was created, and 0.5 mL mitomycin C (0.04%) was applied between the episcleral and conjunctival flap for 5 min [33]. A second scleral flap (3.0 × 2.5 mm) with four-fifths the thickness of the sclera was created and resected with an anterior chamber maintainer. The window (3.0 × 1.0 mm) was opened and peripheral iridectomy was performed. The scleral flap was sutured at 4 points with 10 − 0 nylon and after confirmation of a visible leak around the scleral flap, the conjunctival flap was closed with 10 − 0 nylon. Every subject in this study was prescribed eye drops with 0.5% moxifloxacin, 0.1% betamethasone sodium phosphate, and 0.5% tranilast and continued this treatment course for at least 3 months. If necessary, laser suture lysis was performed. All patients underwent this standardized surgical procedure and postoperative management. Preoperative phakic or pseudo phakic status; use of concomitant cataract surgery; and postoperative complications, including hypotony (< 5 mmHg), choroidal detachment, hypotony maculopathy, and postoperative ocular hypertension (≥ 25 mmHg), were recorded. As we performed twice-daily IOP measurements during hospitalization, we counted cases where IOP was ≥ 25 mmHg in two consecutive measurements if the data were from the hospitalization period. Hypotony maculopathy was defined by the presence of IOP less than 5 mmHg and a disrupted RPE layer in the macular area in the OCT macular map. There were no cases with endophthalmitis or severe reflective changes in the cornea (i.e., > 1 diopter).

### Statistical analysis

Clinical characteristics are shown as the mean ± standard deviation (SD). BCVA or IOP changes after the surgery was analyzed with linear mixed-effect models, setting the subject variable as a random effect. The interaction terms for the group variable (reference, non-BCVA-decline group) and the timepoint variable (reference, baseline) were set as fixed effects. The Shapiro-Wilk test was used to check the normality of continuous variables. In group comparisons, P values were calculated using the Welch T test for parametric data and the Wilcoxon signed-rank test for nonparametric data. For categorical data, P values were calculated using the Pearson chi-square test. Single and multiple logistic regression analyses, adding axial length as an explanatory variable, were performed, and receiver operating characteristic (ROC) curves were calculated for the OCT parameters, the area under the curve, and the optimal cut-off value for predicting BCVA decline. Survival curves were calculated by using all 35 eyes, and estimate the survival probabilities, and P values were calculated using a log-rank test. Calculations were performed with JMP software (Pro version 16.1.0, SAS Institute Japan Inc., Tokyo, Japan) or R software (version 4.1.1, R Foundation, Vienna, Austria). The significance level was set at 5%.

## Results

**Fig. 1:**
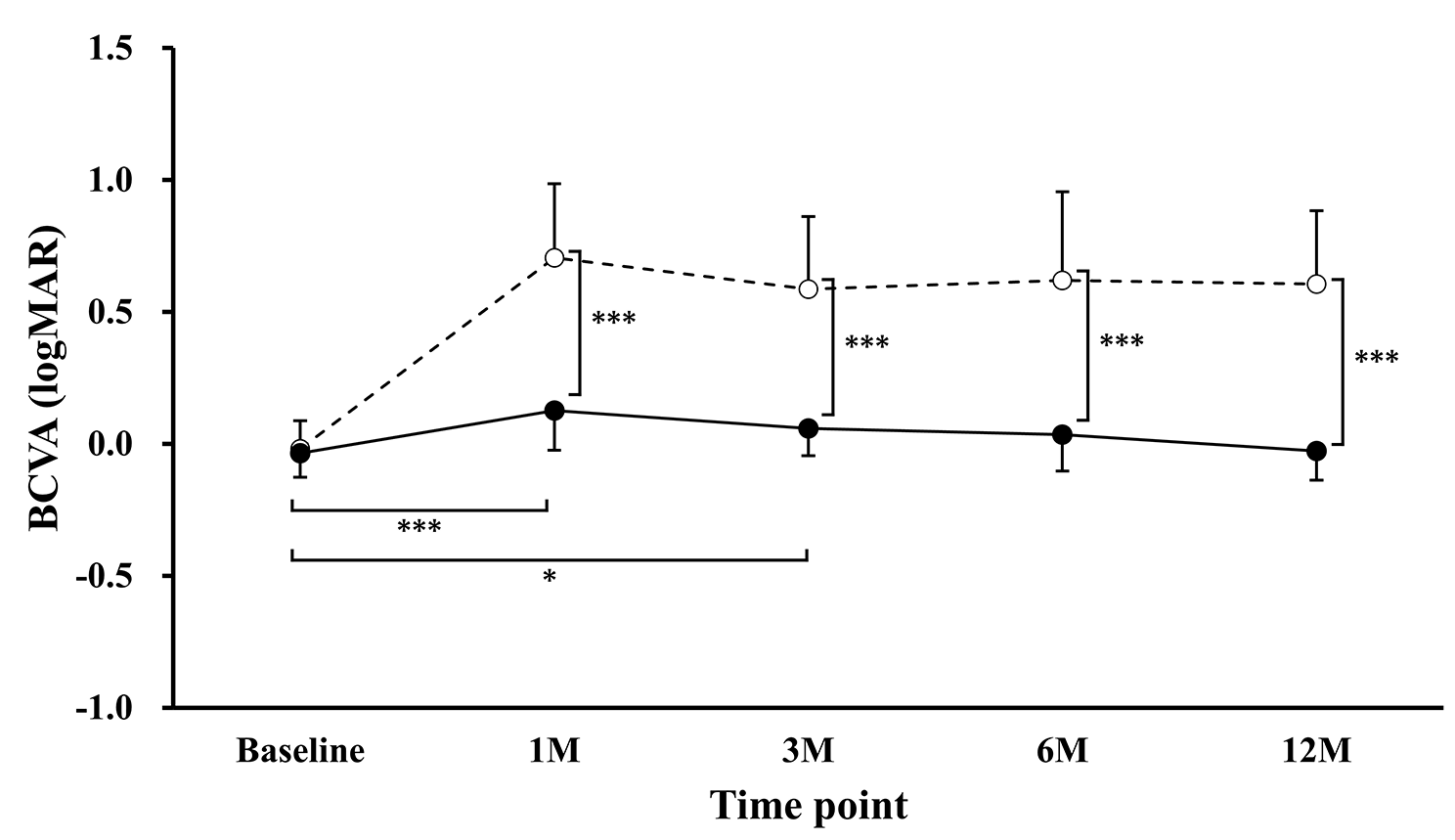
BCVA (logMAR) from baseline to 12 months after trabeculectomy in the non-BCVA-decline (―●―) and BCVA-decline (- -○- -) groups. The vertical axis shows BCVA (logMAR) and the horizontal axis shows the time of examination (month). The BCVA-decline group is indicated by the dotted line. There were no statistically significant differences between the two groups in baseline BCVA, but there were statistically significant differences at 1 month (β = 0.17; P < 0.001; reference, baseline) and 3 months (β = 0.10; P = 0.011; reference, baseline). There were also statistically significant differences in the interaction term in the BCVA-decline group (reference, non-BCVA-decline group) at 1, 3, 6, and 12 months (β = 0.66; P < 0.001, β = 0.57; P < 0.001, β = 0.61; P < 0.001, β = 0.69; P < 0.001, respectively). ^***^: P < 0.001 (linear mixed-effects model). BCVA = best-corrected visual acuity, logMAR = logarithm of the minimal angle of resolution, trab = trabeculectomy

Figure 1 shows the longitudinal change in BCVA (logMAR) from preoperative baseline to 12 months after trab in groups without BCVA decline (N = 24) and with BCVA decline (N = 11). There were statistically significant differences at 1 month (β = 0.17, P < 0.001; reference, baseline) and 3 months (β = 0.10, P = 0.011; reference, baseline). There were also statistically significant differences in the interaction term in the BCVA-decline group (reference, non-BCVA-decline group) at 1, 3, 6, and 12 months (β = 0.66, P < 0.001; β = 0.57, P < 0.001; β = 0.61, P < 0.001; β = 0.69, P < 0.001, respectively).

**Fig. 2:**
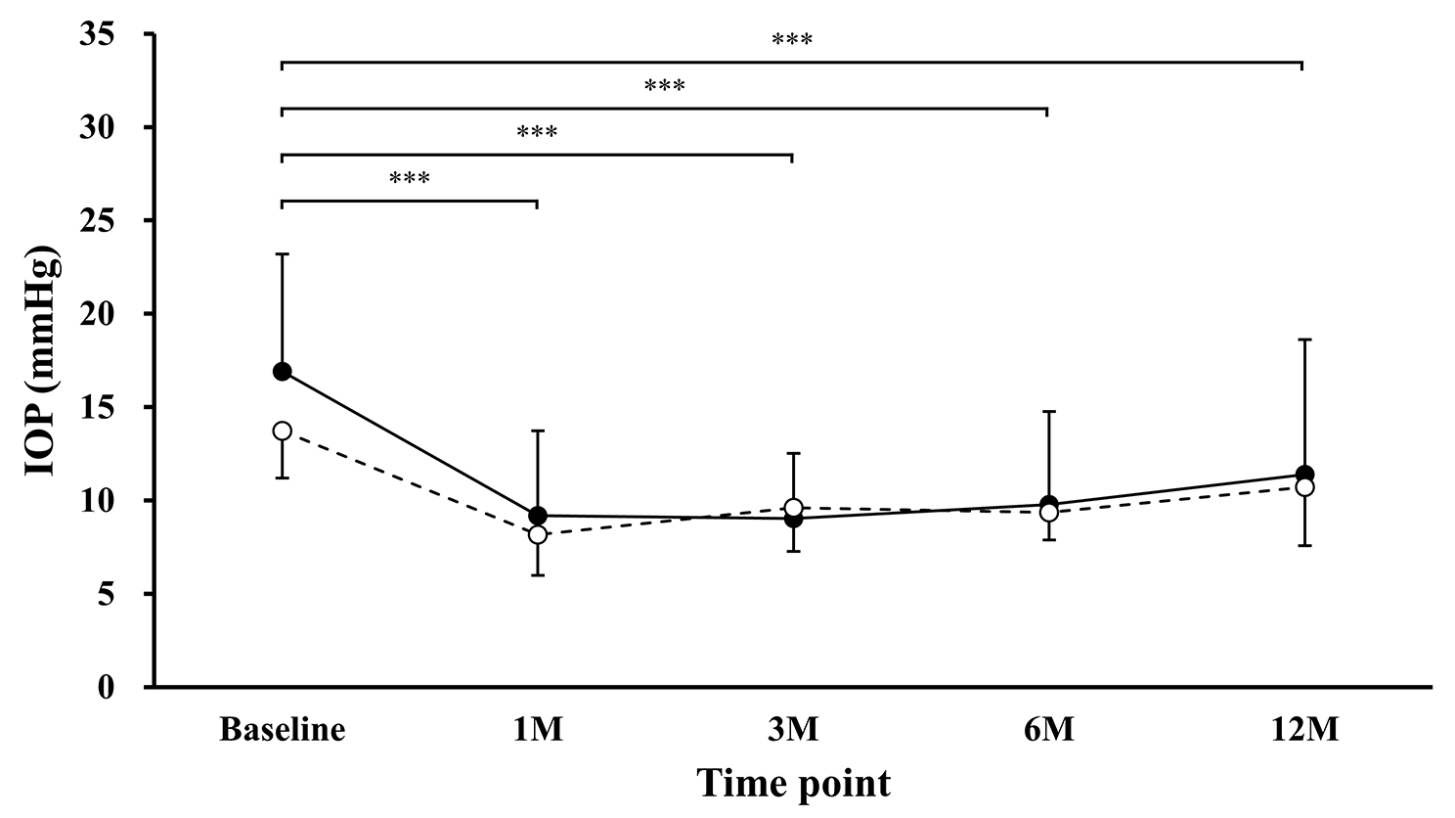
Intraocular pressure (IOP) change from baseline to 12 months after trab in the non-BCVA-decline (―●―) and BCVA-decline (- -○- -) groups. The vertical axis shows IOP (mmHg) and the horizontal axis shows the timepoints. There was a statistically significant difference in IOP at each time point versus baseline. Note that there were no significant differences in the group variable or interaction term between the time point variable and the group variable. ^***^: P < 0.001 (linear mixed-effects model). BCVA = best-corrected visual acuity, trab = trabeculectomy, IOP = intraocular pressure

Figure 2 shows IOP from preoperative baseline to 12 months after trab in the non-BCVA-decline and BCVA-decline groups. There was a statistically significant difference in IOP at each time point (1 M: β = -8.06, 3 M: β = -8.14, 6 M: β = -7.27, 12 M: β = -5.58; all: P < 0.001; reference, baseline). However, there were no statistically significant differences in the interaction term in the BCVA-decline group (reference, non-BCVA-decline group) at 1, 3, 6, or 12 months (β = 1.68, P = 0.356; β = 3.48, P = 0.058; β = 2.64, P = 0.149; β = 2.08, P = 0.254, respectively).

**Fig. 3:**
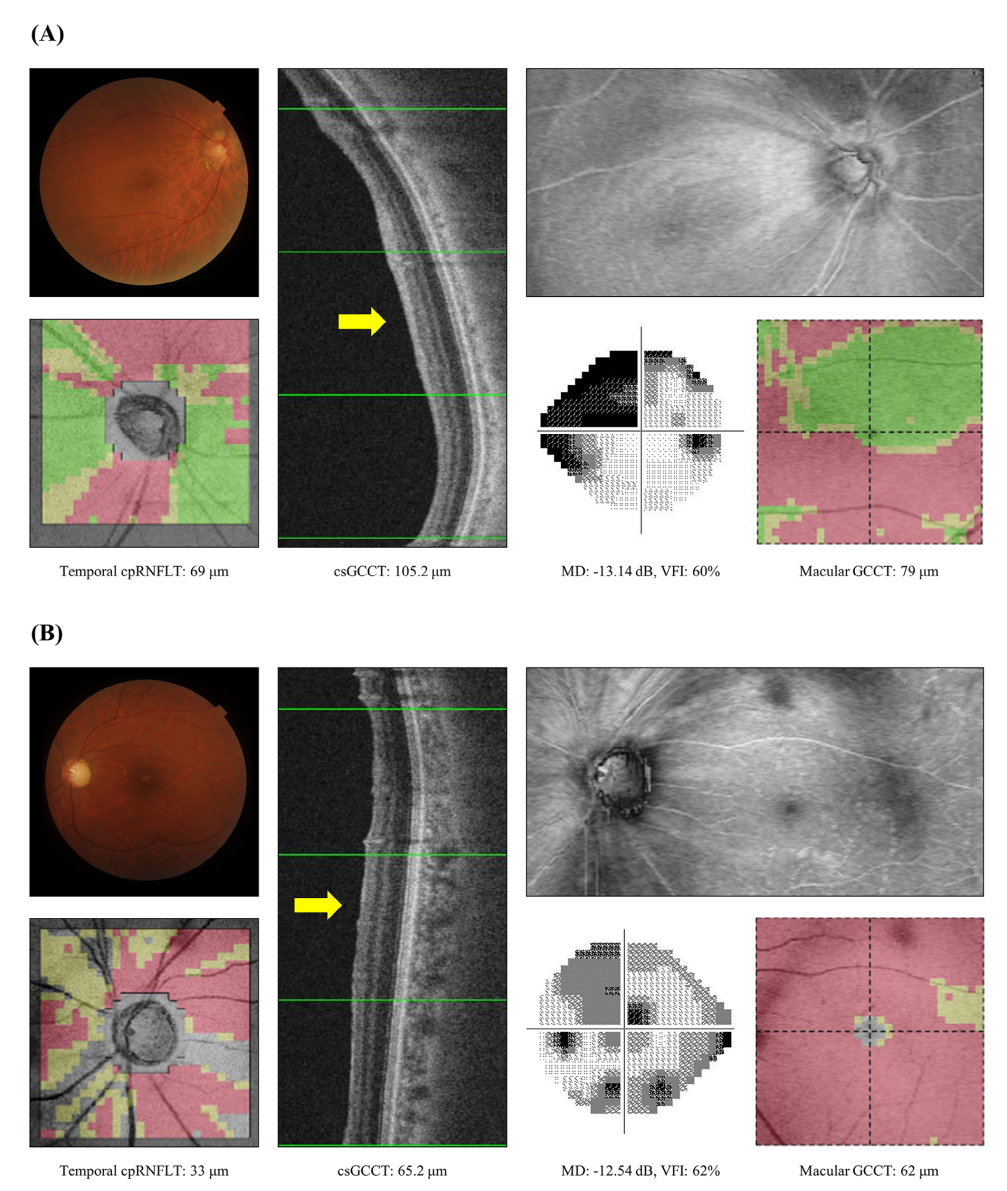
Representative cases from the non-BCVA-decline group and the BCVA-decline group. (**A**): Representative case from the non-BCVA-decline group. All images were obtained from the right eye of a patient with primary open-angle glaucoma. BCVA in the better eye was 20/25 preoperatively and 20/16 postoperatively. IOP was 16 mmHg, mean deviation was − 13.14 dB, csGCCT was 105.2 μm, macular GCCT was 79 μm, temporal cpRNFLT was 69 μm, and VFI was 60% preoperatively. (**B**): Representative case from the BCVA-decline group. All images were obtained from the left eye of a patient with normal-tension glaucoma. BCVA was 20/25 preoperatively and 20/50 postoperatively, IOP was 11 mmHg, mean deviation was − 12.54 dB, csGCCT was 65.2 μm, macular GCCT was 62 μm, temporal cpRNFLT was 33 μm, and VFI was 62% preoperatively. BCVA = best-corrected visual acuity, dB = decibel, IOP = intraocular pressure, MD = mean deviation, GCCT = ganglion cell complex thickness, cpRNFLT = circumpapillary retinal nerve fiber layer thickness, VFI = visual field index

Figure 3 shows representative cases of non-BCVA decline (A) and BCVA decline (B). The non-BCVA-decline case was a patient with POAG in the right eye; BCVA in the better eye was 20 / 25 preoperatively and 20 / 16 postoperatively, and preoperative IOP was 16 mmHg. Preoperative MD was − 13.14 dB and VFI was 60%. The preoperative OCT parameters were as follows: temporal cpRNFLT 69 μm, csGCCT 105.2 μm, and macular GCCT 79 μm. The BCVA-decline case was a patient with NTG in the left eye; BCVA was 20/25 preoperatively and 20/50 postoperatively, and preoperative IOP was 11 mmHg. Preoperative MD was − 12.54 dB and VFI was 62%. The preoperative OCT parameters were as follows: temporal cpRNFLT 33 μm, csGCCT 65.2 μm, and macular GCCT 62 μm.

**Fig. 4:**
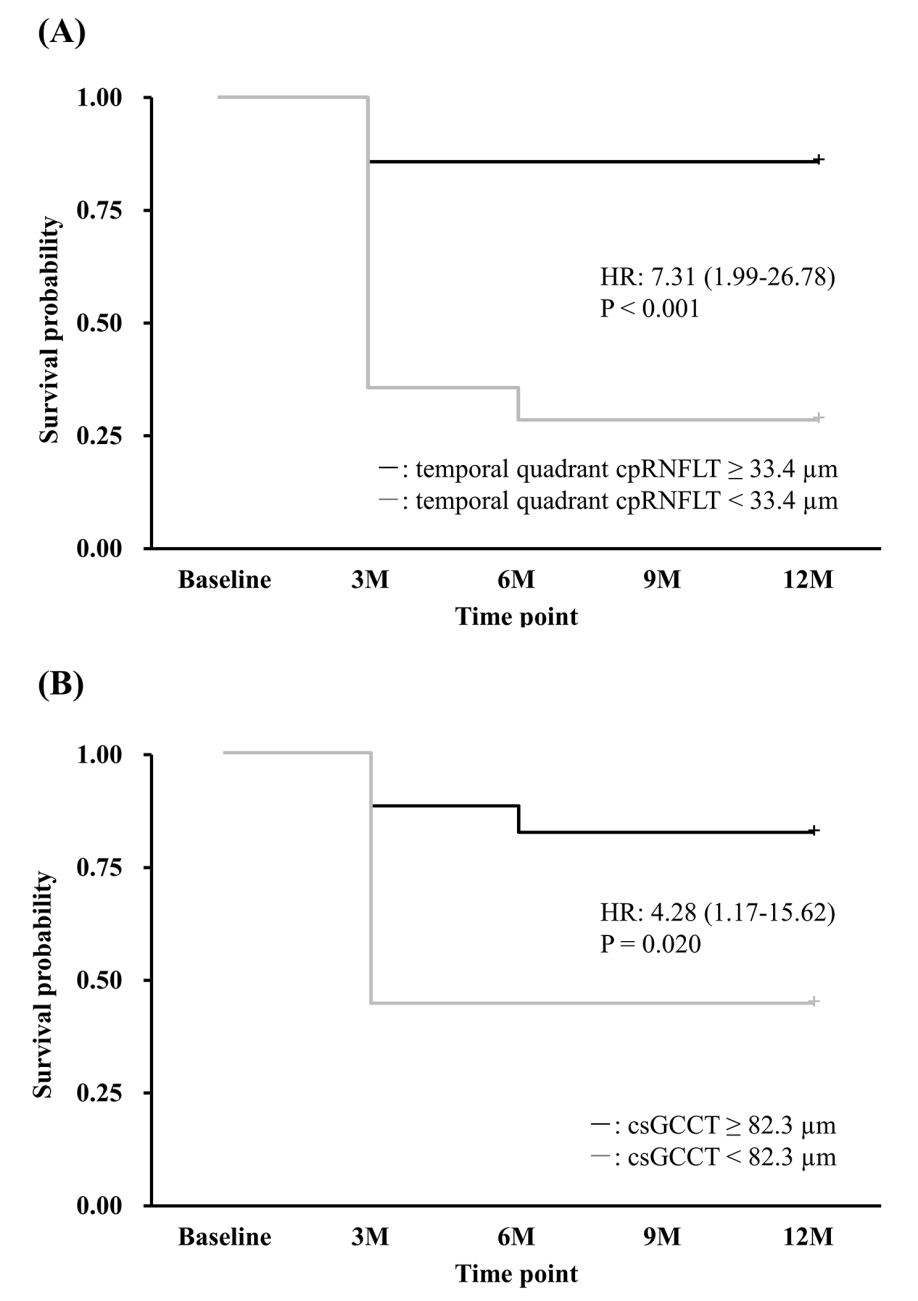
Kaplan–Meier survival curves for significant BCVA decline at 12 months. “Death” was defined as two consecutive findings of vision loss. In Fig. 4A, the black line shows cases with temporal quadrant cpRNFLT no less than the cutoff of 33.4 μm, and the gray line indicates cases with temporal quadrant cpRNFLT below the cutoff. The probability of significant BCVA decline at 12 months was 14.3% and 71.4%, respectively, in these groups (P value < 0.001). In Fig. 4B, the black line indicates cases with csGCCT no less than the cutoff of 82.3 μm, and the gray line indicates cases with csGCCT below the cutoff. The probability of significant BCVA decline at 12 months was 17.6% and 55.6%, respectively, in these groups (P value = 0.020). BCVA = best-corrected visual acuity, cpRNFLT = circumpapillary retinal nerve fiber layer thickness, csGCCT = ganglion cell complex thickness in a central strip between the disc and fovea, HR = hazard ratio

Figure 4 shows the Kaplan–Meier survival curves for significant BCVA decline at 12 months. Since there were some cases in which vision loss occurred and then recovered, “death” was defined for the survival analysis as two consecutive findings of vision loss, and a log-rank test was performed. For groups above and below the cutoff value for temporal quadrant cpRNFLT, the probability of significant BCVA decline at 12 months was 14.3% and 71.4%, respectively (P < 0.001; Fig. 4A), and for groups above and below the cutoff value for csGCCT, the probability was 17.6% and 55.6%, respectively (P = 0.020; Fig. 4B**).**

We compared the background clinical parameters in the non-BCVA-decline and BCVA-decline groups and found no significant differences in age, gender, glaucoma subtypes, complication with hypertension or diabetes mellitus, preoperative IOP, or percentage reduction in IOP at 12 months against preoperative baseline, CCT, MD, or VFI. We found a significant difference in axial length: the non-BCVA-decline group had shorter axial length than the BCVA-decline group (25.2 ± 0.7 mm vs. 26.6 ± 1.0 mm, P = 0.049; shown in Table 1). As shown in Table 2, we also found that there was no significant difference between the two groups in the frequency of postoperative complications, including hypotony (12 / 24 cases vs. 7 / 11 cases, P = 0.452), choroidal detachment (1 / 24 cases vs. 2 / 11 cases, P = 0.216), hypotony maculopathy (3 / 24 cases vs. 3 / 11 cases, P = 0.282), or postoperative hypertension (5 / 24 cases vs. 1 / 11 cases, P = 0.643).

**Table 1:** Comparison of clinical parameters between the non-BCVA-decline and BCVA-decline groups

Variables	Non-BCVA-decline group	BCVA-decline group	P value
	(N = 24)	(N = 11)	
Age (years)	63.7 ± 4.0	63.4 ± 6.0	0.709^b^
Sex (male / female)	15 / 9	6 / 5	0.760^a^
Hypertension (n)	6	5	0.252^c^
Diabetes mellitus (n)	3	2	0.647^c^
Baseline IOP (mmHg)	17.0 ± 2.0	13.7 ± 3.1	0.100
Reduction in IOP (%)	30.0 ± 13.5	18.1 ± 19.2	0.320^b^
CCT (µm)	511.3 ± 12.4	510.5 ± 18.0	0.683^b^
Axial length (mm)	25.2 ± 0.7	26.6 ± 1.0	0.049^b*^
MD (dB)	-18.2 ± 2.3	-18.1 ± 3.4	0.749^b^
VFI (%)	44.8 ± 8.2	47.7 ± 12.4	0.810^b^
TD-central (dB)	-17.5 ± 3.2	-24.6 ± 5.1	0.045^b*^
Split fixation (n)	19	9	0.644^c^
Glaucoma subtypes (NTG/POAG/PE) (n)	8 / 15 / 1	7 / 4 / 0	0.181^c^
Preoperative phakic eyes (n)	13	8	0.298^a^
Combined cataract surgery (n)	3	2	0.656^a^

BCVA = best-corrected visual acuity; CCT = central corneal thickness; IOP = intraocular pressure; MD = mean deviation; NTG = normal-tension glaucoma; PE = pseudo-exfoliation; POAG = primary open-angle glaucoma; TD = total deviation; VFI = visual field index

Percentage reduction in IOP indicates the reduction rate of IOP 12 months after the surgery against the baseline

Unmarked P values: Welch t test; ^a^ Pearson chi-square test; ^b^ Wilcoxon rank sum test; ^c^ Fisher exact test.

^*^ indicates statistical significance

**Table 2:** Comparison of postoperative complications in non-BCVA-decline and BCVA-decline groups

Complications	Non-BCVA-decline group	BCVA-decline group	P value
	(N = 24)	(N = 11)	
Hypotony (n)	12	7	0.452^a^
Choroidal detachment (n)	1	2	0.216^b^
Hypotony maculopathy (n)	3	3	0.282^a^
Postoperative hypertension (n)	5	1	0.643^b^

BCVA = best-corrected visual acuity. P value: ^a^ Pearson chi-square test; ^b^ Fisher exact test

A comparison of preoperative OCT and LSFG parameters in the BCVA-decline and non-BCVA-decline groups showed that the decline group had significantly lower temporal quadrant cpRNFLT (27.7 ± 8.0 vs. 45.1 ± 5.3 μm, P < 0.001), middle csGCCT (72.4 ± 7.7 vs. 87.5 ± 5.1 μm, P = 0.002), and temporal quadrant MT (5.0 ± 1.1 vs. 6.7 ± 0.8 μm, P = 0.018), but that macular GCCT was not significantly different (Table 3). A single logistic regression analysis for discriminating the BCVA-decline group from the non-BCVA-decline group by preoperative structural or BF parameters showed that the largest AUC for the structural parameters was 0.83 for the temporal quadrant cpRNFLT (P < 0.001, cut-off value: 33.4 μm). The next largest AUCs were 0.82 for middle csGCCT (P < 0.001, cut-off value: 82.3 μm) and 0.76 for temporal quadrant MT (P = 0.007, cut-off value: 5.2 AU) (Table 4). AUC was 0.70 for TD-central (P = 0.024, cut-off value: -23.0 dB). Next, we used a multiple logistic regression analysis for these parameters, adding axial length as an explanatory variable (Table 5). The AUCs for temporal quadrant cpRNFLT, middle csGCCT, and TD-central were as follows: AUC = 0.90, P < 0.001; AUC = 0.88, P < 0.001; and AUC = 0.82, P = 0.005).

**Table 3:** Comparison of OCT- and LSFG-derived parameters in the non-BCVA-decline and BCVA-decline groups

Variables	Quadrant	Non-BCVA-decline group	BCVA-decline group	P value
		(N = 24)	(N = 11)	
***cpRNFLT (µm)***				
	Total	56.5 ± 5.4	45.6 ± 8.3	0.088^a^
	Superior	67.1 ± 7.8	58.1 ± 11.8	0.256^a^
	Nasal	60.2 ± 9.2	48.5 ± 13.4	0.139
	Inferior	53.8 ± 6.4	48.3 ± 9.4	0.300
	Temporal	45.1 ± 5.3	27.7 ± 8.0	< 0.001^*^
***GCCT*** ***(µm)***				
	Macula	70.0 ± 3.3	67.4 ± 4.8	0.334
***csGCCT (µm)***				
	Superior	76.0 ± 4.7	67.2 ± 7.0	0.033^*^
	Middle	87.5 ± 5.1	72.4 ± 7.7	0.002^*^
	Inferior	69.0 ± 3.7	70.3 ± 5.5	0.596
***MT (AU)***				
	Total	8.6 ± 0.8	7.1 ± 1.2	0.057^a^
	Superior	8.8 ± 0.9	7.5 ± 1.3	0.057^a^
	Nasal	11.2 ± 1.2	9.6 ± 1.7	0.145^a^
	Inferior	9.0 ± 0.9	7.5 ± 1.4	0.073^a^
	Temporal	6.7 ± 0.8	5.0 ± 1.1	0.018^a*^

Unmarked P values: Welch t test; ^a^ Wilcoxon rank sum test; ^*^ indicates statistical significance

BCVA = best-corrected visual acuity; cs = central strip; cpRNFLT = circumpapillary retinal nerve fiber layer thickness, GCCT = ganglion cell complex thickness; LSFG = laser speckle flowgraphy; MT = tissue-area mean blur rate; OCT = optical coherence tomography

**Table 4:** ORs and AUCs for each variable for differentiating the BCVA-decline group from the non-BCVA-decline group

Variables	Quadrant	OR (95% CI)	P value	AUC (95% CI)	Cut-off value
***cpRNFLT***					
	Total	1.07 (1.00–1.14)	0.028^*^	0.68 (0.46–0.86)	46.2 μm
	Superior	1.03 (0.99–1.08)	0.135	0.64 (0.42–0.80)	59.9 μm
	Nasal	1.04 (0.99–1.08)	0.092	0.61 (0.38–0.80)	38.8 μm
	Inferior	1.02 (0.98–1.08)	0.288	0.56 ( 0.31–0.78)	41.1 μm
	Temporal	1.18 (1.02–1.35)	< 0.001^*^	0.83 (0.65–0.93)	33.4 μm
***GCCT***					
	Macula	1.05 (0.95–1.16)	0.306	0.59 (0.38–0.77)	76.3 μm
***csGCCT***					
	Superior	1.09 (1.00–1.18)	0.021^*^	0.65 (0.46–0.80)	75.7 μm
	Middle	1.13 (1.03–1.24)	< 0.001^*^	0.82 (0.64–0.92)	82.3 μm
	Inferior	0.98 (0.90–1.06)	0.587	0.55 (0.35–0.74)	63.6 μm
***MT***					
	Total	1.49 (1.00–2.22)	0.030^*^	0.71 (0.49–0.87)	7.4 AU
	Superior	1.36 (0.95–1.95)	0.070	0.71 (0.47–0.88)	7.4 AU
	Nasal	1.22 (0.95–1.58)	0.101	0.66 (0.40–0.85)	9.9 AU
	Inferior	1.4 (0.97–2.01)	0.049	0.70 (0.48–0.85)	7.3 AU
	Temporal	1.85 (1.04–3.29)	0.007^*^	0.76 (0.56–0.88)	5.2 AU
**TD-central**		1.13 (0.79–0.99)	0.024^*^	0.70 (0.51–0.86)	-23.0 dB

BCVA = best-corrected visual acuity; cpRNFLT = circumpapillary retinal nerve fiber layer thickness; CI = confidence interval; GCCT = ganglion cell complex thickness; CS = central strip; MT = tissue-area mean blur rate; OR = odds ratio; TD = total deviation

ORs and P values: logistic regression analysis; ^*^ indicates statistical significance; AUC indicates the area under the receiver operating characteristic curve in a logistic regression model

**Table 5:** ORs and AUCs for each variable for differentiating the BCVA-decline group from the non-BCVA-decline group, with axial length as an additional explanatory variable

Variables	Quadrant	OR (95% CI)	P value	AUC (95% CI)
***cpRNFLT***				
	Total	1.08 (0.59–1.16)	0.029^*^	0.79 (0.59–0.91)
	Superior	1.02 (0.98–1.07)	0.228	0.74 (0.51–0.88)
	Nasal	1.05 (0.99–1.11)	0.048	0.78 (0.57–0.90)
	Inferior	1.02 (0.97–1.07)	0.340	0.73 (0.53–0.87)
	Temporal	1.21 (1.00–1.46)	< 0.001^*^	0.90 (0.73–0.97)
***GCCT***				
	Total	1.09 (0.96–1.22)	0.139	0.74 (0.53–0.88)
***csGCCT***				
	Superior	1.10 (1.00–1.20)	0.022^*^	0.80 (0.60–0.92)
	Middle	1.14 (1.02–1.26)	< 0.001^*^	0.88 (0.71–0.96)
	Inferior	1.00 (0.92–1.10)	0.933	0.71 (0.50–0.86)
***MT***				
	Total	1.4 (0.92–2.12)	0.098	0.79 (0.57–0.92)
	Superior	1.22 (0.83–1.82)	0.294	0.75 (0.53–0.89)
	Nasal	1.18 (0.90–1.54)	0.218	0.78 (0.55–0.91)
	Inferior	1.35 (0.93–1.96)	0.097	0.77 (0.54–0.90)
	Temporal	1.80 (0.97–3.32)	0.023^*^	0.81 (0.62–0.92)
***TD-central***		1.14 (0.74–0.99)	0.005^*^	0.82 (0.61–0.93)

BCVA = best-corrected visual acuity; cpRNFLT = circumpapillary retinal nerve fiber layer thickness; CI = confidence interval; GCCT = ganglion cell complex thickness; CS = central strip; MT = tissue-area mean blur rate; OR = odds ratio; TD = total deviation

ORs and P values: multivariable logistic regression analysis; ^*^ indicates statistical significance; AUC indicates the area under the receiver operating characteristic curve in a multiple logistic regression model

We also report the difference in macular clockwise sector GCCT in Supplementary Table S1. We observed a tendency toward thinning in the GCC in the sector corresponding to the optic disc macular fibers at 9 o’clock in the BCVA-decline group (P = 0.059), though it did not reach statistical significance.

There was no clear deterioration in TD-central or temporal cpRNFLT in the BCVA-decline group during this period (N = 9 of 11, preoperative TD-central: -23.9 ± 5.1 dB vs. postoperative TD-central: -24.8 ± 4.7 dB, P = 0.670; N = 6 of 11, preoperative temporal cpRNFLT: 32.1 ± 7.2 μm vs. postoperative temporal cpRNFLT: 37.6 ± 7.0, P = 0.147; paired t-test).

## Discussion

Postoperative BCVA decline is one of the most serious complications of trab, and it is still difficult to predict. Ophthalmologists experience difficulties in determining indications for trab, especially for patients with good preoperative BCVA. Thus, in this study, we explored clinical parameters that can predict BCVA decline after trab. We recruited OAG patients who underwent trab and had good preoperative BCVA (decimal BCVA ≥ 0.7) and analyzed the correlations between preoperative parameters from SS-OCT or LSFG and the incidence of BCVA decline. We found that 11 cases (31.4%) experienced BCVA decline and that longer axial length was a significant associated factor, but that percentage reduction in IOP, severity of visual field defects, and the frequency of complications, including hypotony, choroidal detachment, the condition of the IOL, and hypotony maculopathy were not. We also found that temporal-quadrant cpRNFLT (AUC = 0.83, cut-off value: 33.4 μm) and middle csGCCT (AUC = 0.82, cut-off value: 82.3 μm) were significant predictors of BCVA decline after trab, and a survival analysis with Kaplan–Meier curves showed that groups with values above the cutoffs had significantly higher survival probability. Furthermore, the AUC improved to 0.90 and 0.88 for temporal quadrant cpRNFLT and middle csGCCT, respectively in the multiple logistic regression models. Therefore, the main finding of this study is that paying attention to PMB structure may help improve decision-making concerning surgical intervention for physicians and patients and help predict the risk of BCVA decline after trab.

In this study, we retrospectively investigated BCVA decline 12 months after trab in 35 OAG patients. We found that VA decline occurred in 11 patients (31.4%). This frequency of vision loss is within the range of values reported in previous reports, which ranged from 0 to 32%; thus, we consider it reasonable [17, 21, 34, 35]. Kashiwagi et al. reported that in 694 eyes of 694 glaucoma patients, 28.3% (95% CI: 24.5–32.0%) were judged to have visual acuity loss, and also reported that in glaucoma subtypes, poor preoperative BCVA and MD and postoperative complications were the main risk factors for visual impairment [35]. However, in the current study, we found that the frequency of postoperative complications did not differ between the groups with and without BCVA decline, and that glaucoma patients with preoperative PMB thinning tended to undergo BCVA decline. To the best of our knowledge, this is the first study to show the risk factors for significant BCVA decline after trab in OAG patients with good preoperative BCVA. We believe that the current study will be useful for physicians and patients considering whether to undergo trab.

We found that long axial length was also a risk factor for BCVA decline after trab (AUC = 0.72). In Asia, NTG is the most common type of OAG [36]. Myopia is a significant risk factor for NTG [37], and changes in the macular area tend to occur in patients with NTG. In this study, a logistic analysis showed that AUC improved after adjusting for axial length, as did the ability to predict BCVA decline. The loss of cpRNFLT is strongly associated with an increase in axial length, making it often difficult to distinguish whether the loss of RNFLT is due to myopia or glaucoma [38, 39]. Nowadays, as many societies are aging, the number of glaucoma patients is expected to increase; myopia in children has also become a problem in recent years [40]. These trends may result in an increased number of glaucoma patients who also have myopia in the future. The goal of trab is to decrease IOP and maintain the QOL of glaucoma patients. Therefore, even if IOP is lowered after trab, the surgery cannot be said to have a good outcome if BCVA decline occurs. More accurate algorithms and multimodal measurement techniques to predict BCVA decline after trab would be valuable additions to future longitudinal glaucoma care.

In addition to axial length elongation, impaired ONH-tissue BF was also a significant predictor of BCVA decline after trab. The pathophysiology of BCVA decline depends on damage in the PMB. We previously reported that in myopic glaucoma, peripapillary choroidal microvasculature dropout and impaired BF in the adjacent temporal ONH-tissue contributed to PMB thinning and central visual field defects [32, 41] which is consistent with the current findings. In this study, preoperative PMB thickness in the BCVA-decline group was already below the cut-off value of 88.6 μm that we reported for BCVA loss [25], indicating that there was no redundancy in neuron fibers. The ischemia-reperfusion cycle that is usually tolerable during trab might thus have led to the irreversible BCVA decline we observed in this study, as these eyes were already in a damaged neuronal state. Another possible cause of BCVA decline is direct additional damage to the already damaged optic nerve fiber caused by the position of the lamina cribrosa changing due to fluctuations in IOP during the operation [42]; this fluctuation is normally tolerable. Furthermore, surgical intervention causes significant stress to the already damaged nerve fibers, leading to inflammatory changes and irreversible retinal ganglion cell death, which could be a cause of BCVA decline after trab. Thus, significant damage to the PMB, manifesting as OCT findings of a thin PMB, and low BF in the temporal ONH-tissue, in addition to axial length elongation, are risk factors for BCVA decline that are measurable with objective instruments. These findings might be important information to provide patients for informed consent before they choose trab. Instruments to measure OBF are fewer than those for measuring OCT, and objective assessment of the PMB with OCT is a critical tool in the clinic.

The wipe-out phenomenon, which has an incidence of 0 to 15%, is regarded as a critical problem related to trab for both clinicians and patients due to the critical influence of BCVA on QOL. Previously, the risk of wipe-out was reported to be related to changes in the visual field confined to a central 10-degree island [14], as well as to older age [16], high mean postoperative IOP, and hypotony maculopathy [19]. The Advanced Glaucoma Intervention Study showed that preoperative risk factors for visual acuity loss were better preoperative visual acuity, older age, and less formal education [43]. Another report found that after treatment with the Ex-PRESS shunt (Alcon, Fort Worth, Texas, USA) or trab, mean BCVA was significantly reduced compared to preoperative (P < 0.001) at day 1 in both groups, recovered at 1 month in the Ex-PRESS group, and recovered at 3 months in the trab group [44]. A different group showed that BCVA significantly decreased 1 week to 3 months after trab, but that in contrast, BCVA was stable and underwent no significant decline after Ex-PRESS [45]. Recently, in a study that recruited 453 participants, visual acuity at 24 months was found to be slightly better in a medical management group than in a trab group (P = 0.006) [46]. Thus, when considering indications for surgery in groups at high risk for BCVA decline, minimally invasive glaucoma surgery or medical management are also good choices for glaucoma management. Before we started this study, there were no reports that investigated risk factors for BCVA decline after trab in OAG patients with good preoperative BCVA, which motivated us to design this project.

Here, we found that the assessment of preoperative OCT parameters in areas corresponding to the location of the PMB could effectively predict VA decline 12 months after trab. We found that OCT measurements made in the region of interest involving the PMB were especially useful. Because of its potential to have high reproducibility and its objective nature, OCT is widely used in private clinics [47, 48]. Preoperative quantitative analysis is useful for decision-making concerning surgical indications. We have conducted research to develop an objective assessment of BCVA in glaucoma patients and have demonstrated that the detection of a thinning PMB is important because of its close association with BCVA in glaucoma patients. We have previously shown that temporal cpRNFLT was significantly associated with BCVA [24]. We consider that this correlation between BCVA and the level of damage in the PMB in glaucoma patients is useful for informing speculation as to how much these factors influence each other in glaucoma patients with cataract. We have also explored more efficient methods, such as examinations of the central-strip area, for evaluating the PMB in glaucoma patients [25]. These methods are more useful than standard algorithms that use overall cpRNFLT and macular maps for objectively evaluating BCVA. Recently, we found that nerve fibers related to BCVA pass through the superior area to the macular-disc center line [25]. In this study, we attempted to determine whether assessment of the PMB with OCT is also useful for predicting BCVA after trab. Our findings suggest that the PMB is important for this, and that overall cpRNFLT, temporal quadrant cpRNFLT, and middle csGCCT were effective predictors of BCVA decline. These findings suggest that objective, quantitative preoperative OCT evaluations are a useful part of glaucoma care.

Potential limitations of this study include its retrospective design, small number of patients with BCVA decline, and short follow-up period. Especially for the parameters shown in Table 2 that had a small number of events (N = 1 or 2), it is difficult to make strong statements about statistical significance. Nevertheless, we consider that these complications are unlikely to have made a substantial contribution to the observed decline in BCVA, an assertion we believe we can still make due to the low prevalence of N = 1 or 2. Francis et al. [34] reported that transient vision loss after trab was common, and that it took a mean of 78 days (range, 6 to 720 days) for recovery. During these 12 months, no additional surgery, either for cataract or glaucoma, was needed in our patients. Nevertheless, research into risk factors influencing BCVA after trab needs to focus on the condition of the lens and the incidence of surgical complications. In this study, there was no difference in preoperative phakia or pseudophakia between the patients who did or did not experience BCVA decline after trab (P = 0.298). To exclude the effect of visual acuity improvement due to combined cataract surgery, patients with postoperative visual acuity improvement were excluded from our analysis. In addition, our relatively strict definition of VA decline as the loss of at least three lines of BCVA without the presence of cataract within the 12-month term of the study suggests that lens condition and lens surgery should not have affected BCVA decline in our patients. Furthermore, OCT image quality is as an objective index that can serve as a potential indirect parameter of lens transparency; we were able to compare preoperative and postoperative OCT image quality in 6 of 11 BCVA-decline cases, and we did not observe any significant deterioration (preoperative: 55.8 ± 10.2 AU; postoperative: 51.9 ± 14.4 AU, P = 0.571; paired t-test). Another possibility could be that the natural course of glaucoma progression, which eventually impacts the central visual field, led to a decline in BCVA, regardless of trab. However, considering that there were no differences in the preoperative MD values and the frequency of high postoperative IOP, we suspect that the decline in BCVA was not due to variation in the natural course of glaucoma progression. It would be unusual for the disease state to be completed within the first month and for its progression to halt thereafter, both in terms of cataract progression and the natural course of glaucoma progression. The frequency of other potential complicating factors that could have influenced BCVA, including hypotony, choroidal detachment, hypotony maculopathy, postoperative hypertension, and bullous keratopathy, also did not significantly differ in the two groups in this study, indicating that these factors were also not the cause of BCVA decline. We therefore speculate that the irreversible functional decline in the central part of the retina might have been due to damage from surgery (e.g., intraoperative pressure fluctuations, inflammation, and ischemia-reperfusion), which patients normally tolerate, but might not have been able to in our cases because of the already damaged neurons.

## Conclusion

In conclusion, we found that patients with severe damage in the PMB tended to have worse BCVA after trab. Clinicians should pay close attention to these patients when making decisions on whether to perform trab in OAG patients with good vision.

### Electronic supplementary material

Below is the link to the electronic supplementary material.

Supplementary Material 1

## Data Availability

The datasets generated and/or analyzed during the current study are not publicly available due to the nature of this research, participants of this study did not agree for their data to be shared publicly but are available from the corresponding author on reasonable request.

## List of Abbreviations

BCVA: Best-corrected visual acuity
BF: Blood flow
CCT: Central corneal thickness
cpRNFLT: Circumpapillary retinal nerve fiber layer thickness
csGCCT: Ganglion cell complex thickness in a central strip between the disc and fovea
GCC: Ganglion cell complex
HFA: Humphrey visual field analyzer
IOP: Intraocular pressure
logMAR: Logarithm of the minimum angle of resolution
MD: Mean deviation
MT: Tissue-area mean blur rate
NTG: Normal-tension glaucoma
OAG: Open-angle glaucoma
OBF: Ocular blood flow
ONH: Optic nerve head
PE: Pseudo-exfoliation
PMB: Papillomacular bundle
POAG: Primary open-angle glaucoma
QOL: Quality of life
ROC: Receiver operating characteristic
SITA: Swedish interactive threshold algorithm
trab: Trabeculectomy
VFI: Visual field index
