# Supplementary material for: Predictive potential of optical coherence tomography parameters for the prognosis of decreased visual acuity after trabeculectomy in open-angle glaucoma patients with good vision
Source: BMC Ophthalmol. 2023 Oct 4;23:399. doi: 10.1186/s12886-023-03145-3 (PMC10548727; doi:10.1186/s12886-023-03145-3)
Supplement: Supplementary file 1 — Supplementary Material 1 [file 12886_2023_3145_MOESM1_ESM.pdf]

Table S1. Comparison of OCT-derived parameters in non-BCVA-decline and BCVA-decline groups.

Variables	Macular clockwise sector	Non-BCVA-decline group (N = 24)	BCVA-decline group (N = 11)	P value
GCCT (μm)				
	7 o'clock	62.6 ± 3.3	62.1 ± 5.1	0.718[a]
	8 o'clock	75.9 ± 4.8	70.6 ± 7.3	0.222[a]
	9 o'clock	77.4 ± 5.7	68.3 ± 8.7	0.059[a]
	10 o'clock	76.1± 4.6	71.0 ± 3.9	0.311[a]
	11 o'clock	63.0 ± 3.2	65.1 ± 4.9	0.186[a]

[a] Wilcoxon rank sum test; * indicates statistical significance.

BCVA = best-corrected visual acuity; GCCT = ganglion cell complex thickness;

OCT = optical coherence tomography.
